# Melissopalynological Study, Phenolic Compounds, and Antioxidant Properties of *Heterotrigona itama* Honey from Johor, Malaysia

**DOI:** 10.1155/2020/2529592

**Published:** 2020-06-22

**Authors:** Mahani Majid, Mohammed S. Ellulu, Mohd Fadzelly Abu Bakar

**Affiliations:** ^1^Faculty of Applied Sciences & Technology, Universiti Tun Hussein Onn Malaysia (UTHM), Hab Pendidikan Tinggi Pagoh, KM1, Jalan Panchor, 84600 Muar, Johor, Malaysia; ^2^Johor State Department of Agriculture, Pusat Pentadbiran Kerajaan Negeri Johor, 79000 Iskandar Puteri, Johor, Malaysia; ^3^Department of Clinical Nutrition, Faculty of Applied Medical Sciences, Al-Azhar University of Gaza (AUG), Gaza City 00970, State of Palestine

## Abstract

Six honey samples produced by the stingless bee *Heterotrigona itama* were analyzed for their plant sources, phenolic compositions, and antioxidant activities. The honey samples were acetolyzed and identified microscopically, and the phenolic compounds were identified by using HPLC-DAD. The antioxidant activities were evaluated using three different assays (FRAP, DPPH, and ABTS) by spectrophotometry. The melissopalynological analysis showed that 26 pollen types from 14 plant families were identified in the honey. *Cocos nucifera* and *Rhizophora mucronata* presented as predominant pollen. A total of 6 phenolic acids such as catechin, chlorogenic acid, epicatechin, protocatechuic acid, *p*-coumaric acid, and rutin were identified. *Rhizophora mucronata* honey possessed the highest antioxidant activity in all assays. The result showed the influence of plant sources on the phenolic compounds and the antioxidant properties of stingless bee honey. These findings could be significant contributions for the sustainability of stingless bee industry as well as to promote Malaysian stingless bee honey worldwide.

## 1. Introduction

Honey is the most popular and most recognized natural food produced by both honeybees (*Apis* spp.) and stingless bees (*Trigona* spp.) from nectar and honeydew [1]. Since ancient times, honey was claimed to have desirable composition that it was in use as a superior remedy to treat illness as well as served as food supplements [2]. This natural product is additionally well known for its high therapeutic and nutritional value [3–6]. Generally, honey consists mainly of glucose and fructose sugars and includes about 200 bioactive substances, including minerals, vitamins enzymes, organic acids, proteins, and phytochemicals [7]. It has been documented that plant sources of honey have a significant effect of polyphenolic compounds, including phenolic acids and flavonoids [8].

In Malaysia, 35 species of stingless bees have been documented and bee farming is a new potential industry in agriculture. The introduction of stingless beekeeping in 2004 by the Malaysian Agricultural Research and Development Institute (MARDI) was expected to provide new alternative species that could supplement the current beekeeping projects from *Apis* spp. in terms of honey productions and pollination services [9]. Among the diverse species of stingless bees, only two species namely *Heterotrigona itama* and *Geniotrigona thoracica* have been widely reared for the purpose of commercial honey production. Of these, in the southern part of Malaysia, *H. itama* is a species that can be easily found in forest and were highly sought by beekeepers. Although the body size of *H. itama* is small, it is possible for them to collect many types of nectar and pollen during foraging. The variation in floral sources as well as geographical origins might produce various types of honey with their own uniqueness, which being a marker to differentiate them from one another. Scientific curiosity regarding the origin of products produced by bees, as well as commercial advantages in determining their quality, stimulated research activities using the knowledge of pollen grain morphology as an investigative tool. However, a few melissopalynological studies have been reported especially for stingless bee species in Malaysia.

The complexity of stingless bee honey composition is the major challenge in honey research, thus the profiling and understanding of honey produced from a different region are important. By doing so, the honey from predominant sources gained a lot of interest because of their specific phenolic profile related to the botanical origin [10]. Honey's antioxidant potential depends not just on the existence of total phenolic compounds but also on the existence of flavonoids, which play a significant role in reducing oxidative stress [11]. The existence of phenolic acids and flavonoids contributes to the functional and therapeutic properties of honey. Previous studies showed that there were a good correlation between botanical origin and phenolic compounds such as phenolic acids and flavonoids. However, there are only few studies on biological activity of honey produced by stingless bees available, even though stingless bee honey has a better composition than *Apis* spp. honey [12]. Thus, the exploration for the therapeutic potential of stingless bee honey is needed to fight numerous diseases since there are not many reports in Southeast Asia including Malaysia.

Taking all these aspects into consideration, the present study was carried out to investigate the pollen compositions, phenolic compounds, and the antioxidant activity of honey produced by the stingless bee species (*H. itama*) in Johor, Malaysia.

## 2. Materials and Methods

### 2.1. Collection of Honey Samples

**Figure 1:**
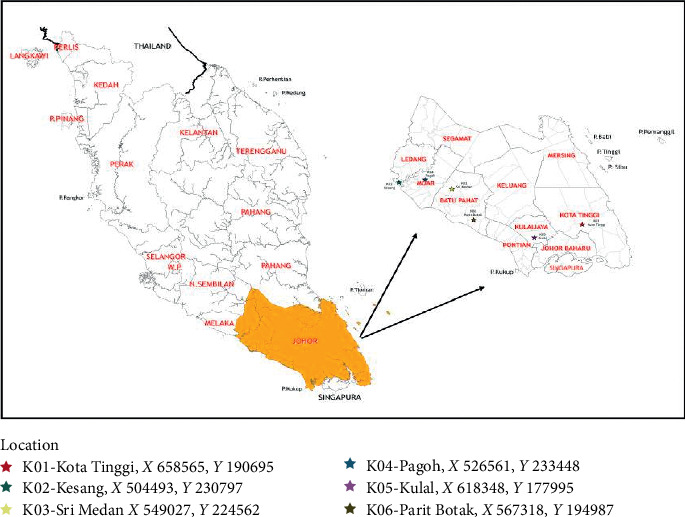
Map of the location of honey samples collected in Johor, Malaysia.

Six honey samples produced by the stingless bee *H. itama* species were used in the study. The identification of *H. itama* species was carried out by Mr. Zakbah bin Mian, an officer cum apiculturist from National Apiary Centre, Department of Agriculture, Malaysia. Honey samples were collected between August and December 2017 in the municipalities of Johor, Malaysia (Figure 1). Within this area, four colonies of *H. itama* were used to collect the honey from each location. Honey pots have been perforated with a sharp tool, and honey was extracted using syringe from individual and collective honey pots. Once collected, the honey samples were sent to the laboratory and processed in sterilized amber glass containers at 4°C in the dark until further analysis.

### 2.2. Melissopalynological Analysis

Characterization and identification of pollen was carried out in accordance with the guidelines provided by the International Bee Botany Commission to ensure the botanical sources. Pollen samples were prepared for the study using an acetolysis process according to Agashe and Rangaswamy [13] with modification. In short, 10 grams of each sample of honey was diluted in 20 mL of distilled water (40°C) and cleared for 10 minutes with centrifugation at 2500 rpm. The supernatant was removed, and 3 mL of glacial acetic acid was applied to the residue, allowing it to stand five minutes until centrifugation and decantation. Then, 1 mL of 10% potassium hydroxide (KOH) was added to the sediment and boiled in 70°C water bath for 5 minutes. This procedure transforms the pollen into light to golden brown in color. After that, the mixture was centrifuged and potassium hydroxide was removed. The residue containing pollen was spread evenly with a micro spatula over an area of 22  mm × 22  mm on the slide. The spread was placed on glycerin jelly and examined under a compound microscope with magnifications of 400× 600×, and 1000×. At least 300 pollen grains were counted from each sample for quantification of the pollen grains. The frequency percentages of the pollen taxa were determined in all samples. The pollen types were allocated to one of the four groups of frequency: predominant pollen (PP, >45%), secondary pollen (SP, 16%–45%), important minor pollen (IP, 3%–15%), and minor pollen (MP, <3%). The pollen identification was achieved by comparing slides of pollen collected directly from plants in the study region with references. Furthermore, the selected palynological literature and monographs have been used according to the approaches of Ibrahim et al. [14], Anthonysamy and Abdullah [15], and Maishihah and Kiew [16].

### 2.3. High-Performance Liquid Chromatography (HPLC) Analysis

Stingless bee honey samples were prepared and extracted using ethyl acetate, as stated by Aljadi and Yusoff [17]. With some modifications in the proposed process of Dimitrova et al. [18], solid-phase extraction technique was used to recover phenolics from honey samples. Dry extracts of honey were dissolved in deionized acidified water (pH 3.5); after that, the dissolved phenolics were absorbed into a preconditioned ISOLUTE C18 column. Five mL of methanol and of acidified water (pH 3.5) were transferred to the preconditioned cartridge in a dropwise flow rate, and phenolic extracts were transferred through the cartridge for efficient adsorption of phenolic compounds. The adsorbed phenolic compounds were eluted from the cartridges at the end of the process by passing 3–5 mL of methanol-water solution (50%, v/v) in a dropwise flow rate. The analyses of HPLC were carried out with modification using Shimadzu (Kyoto, Japan) adapted from Silva et al. [19]. The HPLC was fitted with an automatic Rheodyne 7125i injector with a 20 *μ*L loop and a diode array detector. The columns used were a Shimadzu LC-18 column (4.6 × 250 mm; Supelco, Bellefonte, PA) and a Shimadzu pre-column C-18 ODS. To analyze the phenolic acids, the elution method consisted of 5% formic acid (solvent A) and MeOH (solvent B). The conditions for elution were 0.01–15 min 20%–30% B, 15–20 min 30% B, 20–30 min 30%–40% B, and 40–50 min 100% B, at a flow rate of 0.8 mL/min. All solvents used were of the standard HPLC grade. The wavelength of 280 nm was employed for monitoring. The detection of phenolic compounds was carried out by comparing the retention time with reference levels to the analytes. Phenolic compounds (catechin, chlorogenic acid, epicatechin, *o*-coumaric acid, *p*-coumaric acid, protocatechuic acid, quercetin, and rutin) were obtained from Sigma (St. Louis) and used as standards of reference.

### 2.4. Determination of Antioxidant Activity

#### 2.4.1. DPPH Radical Scavenging Assay

The free radical scavenging activity of the honey samples was determined with modification by the assay 1,1-diphenyl-2-picrylhydrazyl (DPPH), as defined by Abu Bakar et al. [20]. Initially, 0.3 mM of DPPH solution has been prepared by dissolving 0.0118 g of DPPH in methanol (100 mL). Then, dissolving 1 g of honey in methanol to a final concentration of 0.1 g/mL was prepared for honey solution. A 1.5 mL of the methanolic honey solution aliquot was then combined with 1.5 mL of DPPH solution. At 25°C, the reaction mixture was incubated in the dark for 30 min, and the absorbance was measured using a spectrophotometer at 517 nm. Ascorbic acid was used as positive control. The DPPH value was expressed as IC_50_ (mg/mL) and DPPH activity index (RSA), which was expressed as the measurement of the percent inhibition as follows:

(1) $$\begin{array}{c} \mathrm{DPPH radical scavenging activity}\;\left(\%\right)=\frac{{\text{absorbance}}_{\text{control}}-{\text{absorbance}}_{\text{sample}}}{{\text{absorbance}}_{\text{control}}}\times 100. \end{array}$$

#### 2.4.2. ABTS^+^ Cation Radical Scavenging Assay

The ABTS test was carried out according to the methods of Re et al. [21]. The cation radical ABTS^+^ was synthesized by reaction of a solution of 7 mM ABTS with a solution of 2.45 mM potassium persulfate. The mixture was kept in the dark for 16 h at room temperature. The ABTS^+^ solution was then diluted with distilled water until an absorbance of 0.7 was reached at 734 nm. Aliquots of 2.0 mL from the ABTS^+^ solution were applied to the sample solutions diluted in methanol (MeOH) immediately after preparation to achieve final concentrations between 0 and 100 mg/mL. After 10 min, the percentage inhibition was calculated for each concentration at 734 nm and the ABTS^+^ radical scavenging potential (RAS) was determined using the following equation:

(2) $$\begin{array}{c} \mathrm{ABTS radical scaveging activity}\;\left(\%\right)=\frac{{\text{absorbance}}_{\text{control}}-{\text{absorbance}}_{\text{sample}}}{{\text{absorbance}}_{\text{control}}}\times 100. \end{array}$$

#### 2.4.3. Ferric Reducing Antioxidant Power (FRAP) Assay

The reduction in honey samples was calculated with slight modification based on the method defined by Benzie and Strain [22]. The theory of this approach is built on the reduction of a ferric 2,4,6-tripyridyl-s-triazine complex (Fe^3+^-TPTZ) in the presence of antioxidant to its ferrous, colored form (Fe^2+^-TPTZ). The FRAP reagent was prepared by mixing 2.5 mL of a solution of 10 mM TPTZ (2,4,6-tripyridyl-s-triazine) in 40 mM HCL, 2.5 mL of 20 mM FeCl_3_, and 25 mL of a buffer of 0.3 M acetate at 3.6 pH. It was prepared daily and warmed before use until 37°C. An aliquot of 200 *μ*L of honey solution was mixed with 1.5 mL of FRAP reagent, and reagent absorbance was measured spectrophotometrically at 593 nm after 10 min incubation. For the calibration curve, ferrous sulphate (Fe^2+^) was used, and the result were expressed as milligrams of Fe^2+^ equivalent per 100 grams of honey.

### 2.5. Statistical Analysis

Data were analyzed by using the Statistical Package for Social Sciences, version 22.0, software (SPSS Inc., Chicago, IL, USA). The central tendency of variables was presented by the mean ± standard deviation (SD). One-way ANOVA was used to detect the differences between the continuous variables of the plant sources, phenolic compounds, and antioxidant activities. A *P* value of ≤0.05 was considered statistically significant, and level of confidence was 95%.

## 3. Results and Discussion

### 3.1. Melissopalynological Analysis of Honey

Melissopalynological analysis was used to determine the possible botanical sources of honey, where pollen, spores, and hyphae found in honey were identified. This approach has revealed the plant origin and geographical origin of the foraged nectar in the honey and thus the plants that visited by stingless bees and the consistency of honey [23].

The present study showed a wide variability of pollen grains in honey, which represent their plant sources.  Table 1 shows the total 26 pollen types from 14 plant families, which are identified in the honey samples. They comprise several plant types such as herbs, trees, palms, shrubs, mangroves, and flowering plants. Types of pollen that had no proven botanical affinity were called “undetermined.” The high diversity of pollen contained in the honey represents the flora diversity of the area being studied, a feature that favors the production of honey with different properties. The pollen types *Cocos nucifere* (Arecaceae) in K02 honey and *Rhizophora mucronata* (Rhizophoraceae) in K03 honey were identified as predominant pollen, which are considered as unifloral honey. According to Agashe and Caulton [13], unifloral honey is produced mostly from one plant species, accounting for more than 45% of the total pollen content. Meanwhile, K01, K04, K05, and K06 honey contain multiple types of pollen grains at a low density and hence categorized as multifloral honey.

*Rhizophora mucronata* (Rhizophoraceae) and *Cocos nucifera* (Arecaceae) were the predominant pollen type represented 50.0% of all pollen in K03 honey and 66.7% in K02 honey, respectively. The secondary pollen types *Acacia mangium* (Fabaceae), *Robinia pseudoacacia* (Fabaceae), and *Moringa pterygosperma* (Moringaceae) were especially prevalent in K01 honey at 89.8% of all pollen, whereas in K04 and K06 honey, the pollen types *Syzygium jambos* (Myrtaceae), *Cocos nucifera* (Arecaceae), and *Antigonon leptopus* (Polygonaceae) comprised 19.0%, 21.0%, and 20.0% of pollen, respectively. The frequency of pollen in K05 honey was also poorly presented, which only contains the important and minor pollen. Moreover, the pollen types of *Asystasia intrusa* (Acanthaceae), *Mikania cordata* (Asteraceae), *Acacia mangium* (Fabaceae), *Erythrina orientalis* (Fabaceae), and *Cocos nucifera* (Arecaceae) found in more than one honey sample indicate that these pollen types are main attractive sources for bees either for nectar or pollen source.

**Figure 2:**
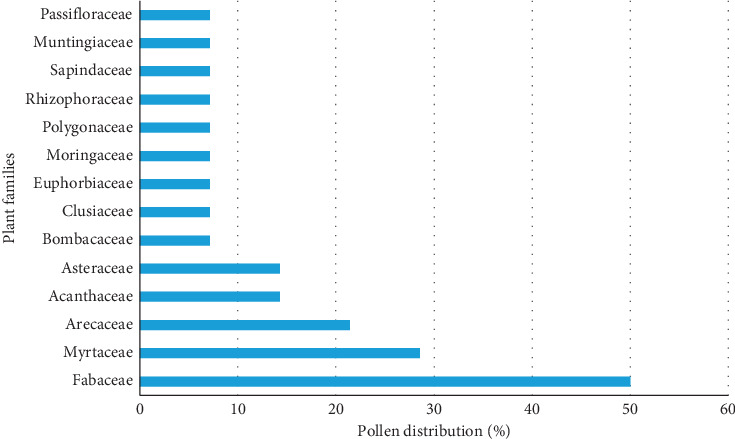
Percentages of pollen grains based on plant families in *H. itama* honey from different plant sources.

Figure 2 shows the pollen distribution in all honey samples based on their plant families. This information is important as it revealed the availability of preferred plant sources for *H. itama* species within the foraging range of the hives. The Fabaceae family was represented by the seven species *Acacia mangium, Robinia pseudoacacia, Peltophorum pterocarpum, Erythrina orientalis, Calliandra portoricensis, Mimosa scabrella,* and *Mimosa bimucronata*, and these species were present in K01, K04, and K05 honey at high frequency. The diversity of Fabaceae as a largest plant family found in tropical rainforest such as Malaysia might attract the *H. itama* bees to visit them either for collecting nectar or pollen that contribute to the honey production. As compared with other countries, the abundance of pollen from Fabaceae in Brazil's pollen analysis proved the importance of this family for bees in the production of honey [24].

Besides Fabaceae, the Myrtaceae family also produced nectar and pollen, which played a good role in honey production, presented in four of the six honey samples. Arecaceae was represented by the three species *Cocos nucifera, Elaeis guineensis,* and *Nypa fruticans,* and these species were present as predominant pollen in K01 honey, important pollen in K03 honey, and minor pollen in K02 honey, respectively. *Cocos nucifera* is an important cultural, perennial plant known as the source of nectar and pollen for bees [25]. While *Eleais guineensis* does not produce nectar, the bees typically only visit it for their pollen [24]. Pollen is one of the significant distinguishing markers of Malaysian honey from imported honeys [15]. *Cocos nucifera* and *Rhizophora mucronata* from the family of Rhizophoraceae were predominant pollen types in K03 honey, which have been recognized as good honey plants [26]. *Bidens pilosa* and *Mikania cordata*, which both belong to Asteraceae, were presented as important and minor pollen types. Moreover, *Antigonan leptopus* (Polygonaceae), which presented as secondary pollen in K06 honey, is a good source of both pollen and nectar for bees. It's a species of flowering plants that have a prolonged blooming period throughout the year [27].

The melissopalynological analysis of the present study revealed a variety of pollen grains collected by *H. itama* bees. However, due to several factors, the bees can change their trophic niche during the year; examples involve floral abundance within a vegetative zone where hives are located, as well as climatic condition. Therefore, the variability of floral sources visited by bees might produce various types of honey, which influence the composition of phytochemical as well as biological activity such antioxidant capacity [10].

### 3.2. Identification of Phenolic Acid by Using HPLC-DAD

Phytochemicals are a wide class of nutraceuticals found in plants that are studied extensively by scientists for their potential for health promotion. Honey is known to have a large variety of phenolic compounds including phenolic acids and flavonoids, which act as antioxidant agents. The analysis of phenolic compounds was seen as a very promising way of investigating honey's floral and geographical origins. All these compounds are mostly the result of secondary plant metabolism and are characterized by several phenolic groups associated with more or less complex structures. The phenolic composition of honey depends mainly on its floral origin, which is suggested as a tool for classification and authentication, in particular for unifloral varieties [28]. Moreover, phenolic is one of the two major medicinal substances present in plants other than terpenoids that fueled honey through its nectar with varying bioactivity.

High-performance liquid chromatography (HPLC) was performed to investigate the phenolic compounds in the honey samples. The HPLC chromatograms show the identification of six phenolic compounds in *H. itama* honey. Chlorogenic acid (CLA) and *p*-coumaric acid (PCA) were present abundant in honey samples. These compounds have been previously reported in monofloral Malaysia honey from *Apis* spp. [29]. In contrast, epicatechin (EP), rutin (RE), catechin (CH), and protocatechuic acid (PTA) were found only in one sample (different specimens of each compound). CLA is commonly found in plant materials such as coffee beans, tea leaves, apples, pulp, peel, and grapes [30]. Therefore, the CLA present in all tested honey samples suggests it is an essential component of stingless bee honey. PCA was present in honey types K02, K03, K04, and K05, which showed strong clinical benefits of honey. PCA is widespread in fruits and vegetables and has positive health effects, such as anti-inflammatory, anticancer, antimicrobials, and antioxidants [31]. In contrast, the compounds of epicatechin (EP), rutin (RE), catechin (CH), and protocatechuic acid (PTA) are present in only one individual sample, meaning that each sample had its own specific phytochemical compounds. However, *o*-coumaric acid (OCA) and quercetin (QH) did not appear in any of honey samples under investigation.

In Malaysia, there is very limited literature reporting the analysis of polyphenolic compounds in stingless bee honey. A study made by Ahmed and Othman [32] reported that *Apis* spp. produced Tualang honey contains gallic acid, trans-cinnamic acid, *p*-coumaric acid, benzoic acid, and caffeic acid. Recently, Ranneh et al. [33] investigated two types of Malaysian honey containing gallic, caffeic, syringic, catechine, cinnamic, *p*-coumaric, apigenin, and 4-hydroxybenzoic as major compounds. Compared with others countries, phenolic compounds such as 3,4-dihydroxy, gallic, vanillic, and trans-cinnamic were detected in *M. subnitida* honey from Brazil by Silva et al. [19]. There are more compounds such as hydroxycinnamic acid, coumaric acid, quercetin, isorhamnetin, methyl luteolin, apigenin, *C*-pentosyl-*C*-hexosyl-apigenin, and kaempferol were identified in *M. beecheii* honey from Cuba [34]. The present study did not detect the described additional phenolic acids, which can be explained by the difference in the origin of the honey, batch, and the method of analysis used.

It may be noted based on the present analysis that the investigation of the phenolic compounds in stingless bee honey has recently been documented and has been growing, with a great variation in their composition. In fact, the location of the apiaries and the geographical origin in which the bees collect the nectar will influence the phenolic composition of the honey [35]. Overall, the polyphenolic compounds detected in the tested honey samples could display promising potent bioactivity and be useful in therapeutic studies.

### 3.3. Antioxidant Activities

Because of several health benefits along with their crucial role in preventing oxidative rancidity of various foods, antioxidants are considered essential bioactive compounds. Three antioxidant assays (DPPH, ABTS, and FRAP) were used in this study to determine the antioxidant capacity of honey from different botanical origins. Each method has its own mechanism and uniqueness. DPPH and ABTS assays were performed to test the water-soluble antioxidant in different methods to determine heterogeneity in most techniques used, whereas FRAP assay was conducted to reflect the evaluation of lipid-soluble antioxidants [17]. Basically, to evaluate honey's radical scavenging potential, the total phenolic and flavonoid contents are directly related to its antioxidant capacity [36]. Previous research showed the total content of phenolic and flavonoid of *H. itama* honey from different botanical origins in methanol extract ranged from 210.11 ± 0.11 to 413.94 ± 0.56 mg GAE/100 g and 179.36 ± 0.12 to 53.90 ± 1.30 mg RE/100 g, respectively [37]. These figures are related to botanical sources, where the main pollen sample presents the highest phenolic and flavonoid content.

#### 3.3.1. DPPH Radical Scavenging Assay

DPPH compound is a stable organic free radical with unpaired valence electron on one atom of the nitrogen bridge. The DPPH radical scavenging method is one of the fastest tests to investigate overall hydrogen/electron-donating behaviour by free radical scavenging of honey antioxidant by reducing DPPH radicals in deep purple color. The DPPH scavenging ability of honey samples from different botanical origin was reported as the percent of DPPH scavenging (% RSA) and expressed in IC_50_ value (mg/mL) in various concentration. Basically, the high DPPH scavenging behaviors are correlated with high antioxidant activity rates. Results obtained in our study showed that in methanolic extract, K03 honey from predominant pollen of *Rhizophora mucronata* and multifloral K04 honey had the lowest IC_50_ value (23.89 mg/mL) and the highest IC_50_ value (130.62 mg/mL), respectively (Table 2). This suggests strong antioxidant activity of K03 honey. Generally, the lower the IC_50_ value, the higher the level of antioxidant potential and *vice versa* [38].

In contrast, the IC_50_ value of tested honey samples in water extract is lower as compared with methanolic extract, and the result showed the same trend. K03 honey displayed the highest level of antioxidant with an IC_50_ value of 34.45 mg/mL, while K04 honey remain the lowest with an IC_50_ value of 170.40 mg/mL. All findings were significantly different (*P* < 0.05). The methanolic extract displayed greater radical scavenging capacity than the water extract, which could be due to the reaction of methanol in various plants and tissues that produce different compound ranges than water. Moreover, this result shows that unifloral honey displayed a higher level of antioxidant potential than the multifloral honey. These findings are highly consistent with those stated by Chua et al. [38], who found the IC_50_ value of Tualang, Acacia, and Gelam honey were 48.90 mg/mL, 29.85 mg/mL, and 15.68 mg/mL, respectively. Meanwhile, a higher IC_50_ value in the range of 3.17 to 8.79 mg/mL has been reported by Pontis et al. [39] for Brazilian honey. In honey, the variation in antioxidant capacities might be due to their diverse plant sources and geographical origins, as well as climate and environmental factors such as temperature, soil composition, and postharvest conditions, which directly affect the composition of nectar and honey production.

#### 3.3.2. ABTS^+^ Cation Radical Scavenging Assay

The principle of ABTS scavenging assay is similar to DPPH scavenging assay, which involve the scavenging activity of free radicals. The sample's antioxidant activity was determined based on the sample's capability to transfer an electron to ABTS radical cation, through the decolorization of the radical cation. The ABTS scavenging ability of the extracts were expressed as the percentages of ABTS scavenging (% RSA). The result showed that all tested honey samples had higher percentages of inhibition in methanolic extract than the water extract. This pattern of the result was in line with DPPH assay because both assays have the same concept, and the results for both assays should also be consistent with similar trend. Table 2 shows that antioxidant content in terms of ABTS inhibition of honey samples in methanol and water extract differed from 18.77 ± 1.06% to 65.02 ± 0.18% and 15.61 ± 3.41% to 65.77 ± 1.24%, respectively. Among the examined honey samples, there was a significant difference (*P* < 0.05) in the scavenger activity of ABTS free radicals. Like DPPH assay, K03 honey and K02 honey again showed the highest percentage of ABTS inhibition with a value of 65.02% and 67.77%, respectively, compared with the other samples, indicating that these unifloral honey types possess high antioxidant potential than multifloral honey. In terms of AEAC, all tested honey varied from 16.29 to 45.30 mg AEAC/100g and 10.94 to 37.47 mg AEAC/100g in methanol and water extract, respectively. Such values were comparable with those recorded in Malaysian raw honey that has 14.23 to 26.64 mg AEAC/100g and Czech honey that has14.15 to 40.71 mg AEAC/100 g [40, 41]. The difference in the amounts of antioxidants depends on several factors, such as plant sources and geographical origins, environmental and climatic conditions, and methods of processing [42].

#### 3.3.3. Ferric Reducing Antioxidant Power (FRAP) Assay

The principle of FRAP assay involves the reduction of ferric 2,4,6-tripyridyl-s-triazine (TPTZ) containing ferric ion (Fe^3+^) to a blue color solution containing ferrous ion (Fe^2+^). This method is a simple and direct test that is widely used in many different substances, including honey, for the determining antioxidant capacity [43]. Among the honey samples analyzed, there was a significant difference (*P* < 0.05) in the scavenger activity of FRAP. The average FRAP value of honey samples were 665.23 ± 396.14 *μ*M Fe (II)/100g in methanolic extract and 581.37 ± 313.47 *μ*M Fe (II)/100g in water extract, respectively, with a significant difference between them (*P* < 0.05). As with DPPH and ABTS assays, the current result showed the same trend where K03 honey exhibited the highest FRAP value of 1401.80 ± 5.18 and 1175.80 ± 2.38 *μ*M Fe (II)/100g in both of methanol and water extract, respectively. K07 honey displayed the lowest FRAP value in methanol extract, while in water extract, the lowest value was exhibited by K04 honey. These results showed a contradiction where K07 honey displayed the lowest value in both DPPH and ABTS assays. This inconsistency may indicate the existence of antioxidant lipid-soluble components, which should be taken into account when assessing the honey's antioxidant potential. Compared with previous research, the current findings were in line with Alves et al. [44] who found the highest FRAP value for samples of dark honey such as locust podshrub (1326.7 *μ*M Fe (II)/100g), arbutus (1321.8 *μ*M Fe (II)/100g), and eucalyptus (953.1 *μ*M Fe (II)/100g). In another study, Kishore et al. [45] reported the FRAP value for Tualang and Gelam honey with 121.89 and 115.61 *μ*M Fe (II)/100g, which is lower than that in our findings. These variations can occur in the honey due to different concentrations of phenolic compounds and flavonoids [46]. Moreover, the plant sources and geographical origins, bee species, as well as climate condition might affect the level of antioxidant capacity of honey.

## 4. Conclusion

Studies have demonstrated that K02 and K03 honey presented high frequency of the *Cocos nucifera* (Arecaceae) and *Rhizophora mucronata* (Rhizophoraceae) pollen types, which classified them as unifloral honey. However, K01, K04, K05, and K06 are considered as multifloral honey, which contain multiple types of pollen grains at low density. Six phenolic compounds were detected in the tested honey samples, which were found in various combinations of chlorogenic acid, epicatechin, *p*-coumaric acid, rutin, catechin, and protocatechuic acid. When evaluating antioxidant function, the highest value of antioxidant assays (DPPH, ABTS, and FRAP) were observed for the *Rhizophora mucronata* honey. This finding has shown that the plant sources can influence honey's phytochemical composition and antioxidant activity. Thus, the present study provides a new insight into food preference of *H. itama* species, which is important for colony sustainability and promotes the meliponiculture industry in this region.

## Figures and Tables

**Table 1:** Pollen spectra and frequency classes of the pollen types in honey produced by *Heterotrigona itama* species in Johor, Malaysia.

Plant family	Pollen type	Honey samples/frequency classes					
		K01	K02	K03	K04	K05	K06
Acanthaceae	*Asystasia intrusa*			(2.0)	(2.0)		(3.0)
	*Avicennia alba*		(14.0)				
Arecaceae	*Elaeis guineensis*			(12.0)			(21.0)
	*Cocos nucifera*		(67.0)				
	*Nypa fruticans*		(3.0)				
Asteraceae	*Bidens pilosa*				(1.0)		(12.0)
	*Mikania cordata*	(2.0)					
Bombacaceae	*Durio zibethinus*					(15.0)	
Clusiaceae	*Garcinia prainiana*	(14.0)					
Euphorbiaceae	*Hevea brasiliensis*				(17.0)		
Fabaceae	*Acacia mangium*	(28.0)				(2.0)	
	*Robinia pseudoacacia*	(34.0)					
	*Peltophorum pterocarpum*				(1.0)		
	*Erythrina orientalis*				(8.0)	(11.0)	
	*Calliandra portoricensis*					(2.0)	
	*Mimosa scabrella*	(7.0)			(13.0)		
	*Mimosa bimucronata*						
Moringaceae	*Moringa pterygosperma*	(28.0)					
Muntingiaceae	*Muntingia calabura*	(20.0)					
Myrtaceae	*Psidium guajava*		(1.0)				
	*Syzygium jambos*				(19.0)	(8.0)	
	*Eucalyptus*						(2.0)
Passifloraceae	*Turnera subulata*						(10.0)
Polygonaceae	*Antigonon leptopus*						(20.0)
Rhizophoraceae	*Rhizophora mucronata*			(50.0)			
Sapindaceae	*Nephelium lappaceum*					(1.0)	
Undetermined				(16.0)	(8.0)	(14.0)	(2.0)
Pollen							

Frequency classes: (i) predominant pollen, PP (>45%); secondary pollen, SP (16%–45%); important minor pollen, IP (3%–15%); minor pollen, MP (<3%).

**Table 2:** Antioxidant activity (DPPH, ABTS, and FRAP) of *H. itama* honey from different plant sources in Johor, Malaysia.

Plant sources	Sample code	Honey extract/antioxidant activity					
		Methanol extract			Water extract		
		DPPH	ABTS	FRAP	DPPH	ABTS	FRAP
		% inhibition	% inhibition	*μ*M Fe (II)/100 g	% inhibition	% inhibition	*μ*M Fe (II)/100 g
Multifloral	K01	40.65 ± 0.89^a^	29.54 ± 0.74^a^	455.80 ± 8.00^a^	44.41 ± 0.52^b^	27.94 ± 1.53^a^	299.80 ± 0.29^a^
Unifloral	K02	66.30 ± 0.27^b^	42.79 ± 0.78^b^	847.80 ± 10.83^a^	63.28 ± 0.44^a^	44.29 ± 2.89^b^	739.80 ± 1.85^b^
Unifloral	K03	74.03 ± 1.44^a^	65.02 ± 0.18^c^	1401.80 ± 5.18^a^	66.78 ± 3.63^a^	65.77 ± 1.24^c^	1175.80 ± 2.38^a^
Multifloral	K04	26.67 ± 0.93^c^	19.07 ± 0.49^d^	309.80 ± 2.23^a^	25.31 ± 0.42^c^	20.12 ± 1.98^d^	269.80 ± 0.34^a^
Multifloral	K05	32.27 ± 0.01^a^	29.73 ± 0.98^e^	425.80 ± 2.39^a^	41.35 ± 0.89^d^	28.98 ± 0.08^e^	375.80 ± 1.81^a^
Multifloral	K06	65.64 ± 0.11^d^	44.89 ± 0.96^f^	931.80 ± 1.12^a^	49.82 ± 0.96^e^	45.35 ± 1.61^f^	749.80 ± 4.57^c^
Tualang	K07	25.72 ± 0.44^e^	18.77 ± 1.06^g^	283.80 ± 0.42^a^	20.97 ± 2.39^c^	15.61 ± 3.41^g^	461.80 ± 1.36^a^

Values are expressed as mean ± standard deviation. Different lowercase letters (a, b, c) in the same column indicate significant differences (*P* < 0.05) between honey from different plant sources with antioxidant activity according to Tukey's test.

## Data Availability

The data sets generated and/or analyzed during the current study are available from the first author on reasonable request.
